# From Structural WBCT Findings to Biomechanical Interpretation in Hallux Valgus: Current Evidence, Limitations, and Clinical Implications

**DOI:** 10.3390/bioengineering13101190

**Published:** 2026-10-13

**Authors:** Hicham Bouredoucen, Pierre-Alexandre Poletti

**Affiliations:** 1 Unit of Musculoskeletal Radiology, Division of Radiology, Department of Imaging and Medical Informatics, Geneva University Hospitals, University of Geneva, 1205 Geneva, Switzerland; 2 Division of Radiology, Department of Imaging and Medical Informatics, Geneva University Hospitals, University of Geneva, 1205 Geneva, Switzerland; pierre-alexandre.poletti@hug.ch

**Keywords:** hallux valgus, weight-bearing computed tomography, first ray, biomechanics, structural imaging, functional interpretation, three-dimensional assessment

## Abstract

Hallux valgus is increasingly characterized using weight-bearing computed tomography (WBCT), which provides three-dimensional information on first-ray alignment and articular relationships under load. However, structural abnormalities identified on static WBCT should not be equated with direct measurements of biomechanics or function. This narrative review examines the evidentiary transition from structural WBCT findings to biomechanical interpretation in hallux valgus. Normal first-ray mechanics provide the reference framework for evaluating altered tarsometatarsal alignment, first-metatarsal rotation, metatarsosesamoid relationships, and medial-column coupling. Throughout the review, three levels of evidence are distinguished: structural relationships directly demonstrated by WBCT, biomechanical interpretations supported by anatomical or experimental evidence, and dynamic functional or prognostic consequences that require independent validation. Within this framework, coexisting three-dimensional abnormalities may be interpreted as mechanically interrelated without implying a universal causal sequence. Postoperative WBCT can characterize residual or corrected structural relationships, but normalization of imaging parameters does not by itself establish restoration of dynamic function or superior clinical outcome. Computational modeling may extend patient-specific geometry toward mechanical estimation, although its validity depends on model assumptions and independent validation. The principal contribution of WBCT is therefore to provide detailed structural information under load that can inform biomechanical hypotheses, while the functional and prognostic significance of these findings remains to be established through dynamic and longitudinal studies.

## 1. Introduction

Hallux valgus (HV) is a complex deformity of the first ray that has traditionally been described using radiographic parameters such as the hallux valgus angle (HVA), intermetatarsal angle (IMA), distal metatarsal articular angle (DMAA), and sesamoid position. These measurements have standardized the assessment of the deformity and enabled its severity to be classified. However, they are based on a two-dimensional representation of a condition involving transverse, sagittal, and rotational components [1,2,3,4,5,6,7]. Recent three-dimensional and weight-bearing imaging studies have further emphasized that rotational and segmental components of HV may be incompletely represented by conventional angular measurements [8].

This limitation is particularly relevant when conventional radiographic measurements are used to infer the three-dimensional configuration of the first ray. The first ray is not an isolated anatomical structure but rather a biomechanical unit comprising the first tarsometatarsal joint, first metatarsal, first metatarsophalangeal joint, phalangeal–sesamoid complex, and medial column [9,10]. Interactions among these structures provide adaptive mobility, stability under load, and efficient force transmission during propulsion [11,12,13]. The metatarsophalangeal–sesamoid complex contributes to load transmission and propulsion, whereas coordinated motion across the medial column contributes to first-ray orientation under load [10]. An assessment based solely on projected angular measurements cannot, therefore, fully characterize the spatial relationships among these components.

The development of weight-bearing computed tomography (WBCT) has enabled three-dimensional analysis of the foot in the standing position. It allows bone alignment, articular relationships, and first-ray configuration to be examined under physiological weight-bearing conditions [14,15,16,17,18]. WBCT-based studies have particularly highlighted the importance of first-metatarsal pronation, first tarsometatarsal joint instability, metatarsosesamoid dissociation, and interactions with the medial column [2,6,19,20,21]. More recent studies have further demonstrated that coronal-plane alignment of the medial column differs among isolated HV, combined HV–flatfoot, and progressive collapsing foot deformity, supporting variable involvement of the medial column across these conditions [22,23]. Collectively, these observations have progressively shifted the conceptual model of HV from a predominantly angular deformity toward a three-dimensional disorder characterized by rotational, translational, and articular abnormalities under load.

This narrative review focuses on the biomechanical interpretation of structural abnormalities identified by WBCT in hallux valgus rather than on providing another comprehensive review of WBCT imaging parameters, which has recently been addressed elsewhere [1]. More specifically, the present review examines the evidentiary transition from static structural WBCT findings to their biomechanical and functional interpretation by distinguishing three levels of evidence: structural relationships directly demonstrated under static weight-bearing conditions, biomechanical interpretations supported by anatomical or experimental evidence, and functional or prognostic consequences that remain hypothetical or incompletely established. Within this framework, the review examines how alterations in first-ray stability, rotational orientation, metatarsosesamoid congruity, and medial-column coupling may be interpreted in relation to normal first-ray function, while critically addressing the limitations of inferring dynamic biomechanics from static imaging. Particular emphasis is placed on the evidentiary boundaries between morphology, biomechanical interpretation, and clinical significance.

## 2. Methods

This narrative review was informed by a structured literature search conducted in PubMed, including MEDLINE-indexed records, and updated on September 12, 2026. The primary search was designed to identify three-dimensional and weight-bearing CT studies relevant to hallux valgus and first-ray relationships. The following search strategy was used: (“hallux valgus”[Title/Abstract] OR “first ray”[Title/Abstract] OR “first metatarsal”[Title/Abstract] OR “first metatarsophalangeal joint”[Title/Abstract] OR “first tarsometatarsal joint”[Title/Abstract] OR sesamoid*[Title/Abstract] OR “medial column”[Title/Abstract]) AND (“weight-bearing computed tomography”[Title/Abstract] OR “weightbearing computed tomography”[Title/Abstract] OR WBCT[Title/Abstract] OR “standing CT”[Title/Abstract] OR “standing computed tomography”[Title/Abstract] OR “cone-beam CT”[Title/Abstract] OR “cone beam computed tomography”[Title/Abstract]). The search covered publications from 1 January 1950 through 12 September 2026 and identified 143 records.

Publications were considered relevant when they addressed hallux valgus or first-ray anatomy and reported three-dimensional or weight-bearing CT findings concerning first-metatarsal rotation, first tarsometatarsal relationships, metatarsosesamoid alignment or congruity, medial-column alignment, postoperative three-dimensional correction, or quantitative WBCT assessment. Publications unrelated to hallux valgus or relevant first-ray relationships, studies of unrelated foot and ankle disorders without transferable relevance to first-ray biomechanics, reports focused exclusively on surgical technique without relevant three-dimensional structural or biomechanical assessment, and conference abstracts without sufficient methodological or outcome information were excluded from the primary WBCT evidence set.

Because the purpose of this narrative review was to interpret structural WBCT findings within a biomechanical framework rather than to perform a systematic effectiveness review, complementary foundational anatomical, experimental, biomechanical, and computational publications were identified through targeted searches and manual screening of the reference lists of relevant original studies and recent reviews. These complementary publications were used when necessary to interpret the mechanical significance of structural WBCT findings and were considered separately from the records identified by the primary WBCT search. Publications in English or French were considered. A total of 55 publications were retained in the final qualitative synthesis, comprising both the primary WBCT evidence and complementary anatomical, experimental, biomechanical, and computational literature used for biomechanical interpretation.

Evidence was synthesized qualitatively according to three predefined interpretative levels: (1) structural relationships directly demonstrated by WBCT under static weight-bearing conditions; (2) biomechanical interpretations supported by anatomical, experimental, or mechanical evidence; and (3) functional, prognostic, or clinical consequences requiring independent validation. Greater interpretative weight was given to findings supported by multiple studies or by convergent anatomical, experimental, and imaging evidence, while isolated observational associations were interpreted cautiously.

## 3. From Structural WBCT Findings to Biomechanical Interpretation

Weight-bearing computed tomography provides three-dimensional information on osseous alignment and articular relationships while the foot is loaded [14,15,16,17,18]. In hallux valgus, it can directly characterize structural features that are incompletely represented by projected radiographic measurements, including first-metatarsal rotational orientation, metatarsosesamoid relationships, and segmental alignment of the medial column [2,4,5,19,20,21]. However, the ability to observe these relationships under load does not make WBCT a direct measurement of biomechanics.

In this review, WBCT is used as an umbrella term for CT systems that acquire three-dimensional foot and ankle images under weight-bearing conditions, including dedicated cone-beam CT systems and other standing CT configurations. These technologies are not necessarily interchangeable: acquisition geometry, spatial resolution, field of view, radiation exposure, reconstruction algorithms, and measurement methodology may differ among systems [14,15,16,17,18]. Accordingly, quantitative values obtained with different WBCT technologies should be compared cautiously, particularly when measurements depend on segmentation, anatomical landmarks, or reference planes.

WBCT measurements are also influenced by the acquisition protocol. Unilateral versus bilateral weight-bearing, the proportion of body weight transferred to each limb, and the position or rotation of the foot, knee, and entire lower limb may alter the three-dimensional configuration recorded during scanning. Foot positioning and loading conditions should therefore be standardized and reported whenever possible. These acquisition-related factors represent an important source of variability and should be considered when comparing measurements across studies or interpreting small differences in individual patients.

Measurement reliability further depends on the definition of anatomical landmarks, segmentation methodology, observer experience, and whether measurements are performed manually or using semi-automated tools. Intraobserver and interobserver reproducibility may therefore vary among parameters and software platforms. Although semi-automated approaches may improve measurement efficiency and reproducibility [24], differences in segmentation and landmark selection remain relevant when interpreting quantitative WBCT data. These methodological sources of variability reinforce the need for standardized acquisition and measurement protocols before individual parameters can be adopted as universal clinical thresholds.

For the purposes of this review, three levels of interpretation should therefore be distinguished. The first comprises structural observations directly derived from WBCT, such as bone orientation, joint alignment, translation, rotation, and articular relationships. The second comprises biomechanical interpretations of these observations based on established anatomical, experimental, or mechanical evidence—for example, the potential consequences of altered metatarsosesamoid congruity for tendon moment arms or of altered first-ray orientation for load transmission. The third comprises dynamic functional or prognostic consequences, including gait performance, plantar-pressure redistribution, recurrence, or patient-reported outcomes. These consequences cannot be inferred directly from static WBCT and require independent functional, longitudinal, or biomechanical evidence.

This distinction is particularly important because loading itself influences the measured configuration of the foot. Differences between weight-bearing and non-weight-bearing acquisitions demonstrate that osseous relationships are load-dependent [25], but static WBCT does not directly quantify force or pressure distribution [26] and, as a static structural examination, does not directly measure muscular activity or dynamic motion. Accordingly, throughout this review, WBCT findings are treated primarily as structural observations under load; biomechanical and functional implications are identified separately according to the supporting evidence.

This interpretative framework shifts the focus from cataloguing three-dimensional measurements to asking how structural abnormalities observed under load relate to the mechanical functions of the first ray. Understanding those relationships first requires consideration of normal first-ray biomechanics.

## 4. Normal Biomechanics of the First Ray Under Load

The first ray constitutes a functional unit comprising the medial cuneiform, first metatarsal, first tarsometatarsal joint, first metatarsophalangeal joint (MTP1), phalangeal–sesamoid complex, and their stabilizing structures [9,10]. For the purposes of this review, its normal function is considered according to four interrelated properties relevant to the interpretation of WBCT findings: controlled mobility, rotational stability, metatarsosesamoid congruity, and propulsive stiffening [10].

### 4.1. Stability with Controlled Mobility

The first ray must combine adaptive mobility with mechanical stability. Excessive rigidity would restrict adaptation to ground reaction forces, whereas uncontrolled mobility would impair force transmission [10,12].

The first tarsometatarsal joint permits small sagittal and rotational movements without loss of congruity [10,20,21]. Physiological stability therefore does not imply an absence of motion but rather the ability to maintain a functional orientation under load. During the terminal phase of gait, this stability allows hallux dorsiflexion and the sesamoid complex to contribute to the formation of an effective propulsive lever [10,27,28]. Importantly, static measurements of first-ray mobility do not necessarily correspond to the motion used during gait, underscoring the distinction between structural assessment under load and dynamic function [29].

### 4.2. Rotational Control of the Medial Column and First Ray

The medial column forms a mechanical chain in which rotational adaptations are distributed across the talus, navicular, medial cuneiform, first metatarsal, and hallux [20]. The physiological orientation of the first ray therefore depends on intersegmental coordination rather than on isolated motion of the first metatarsal.

**Figure 1:**
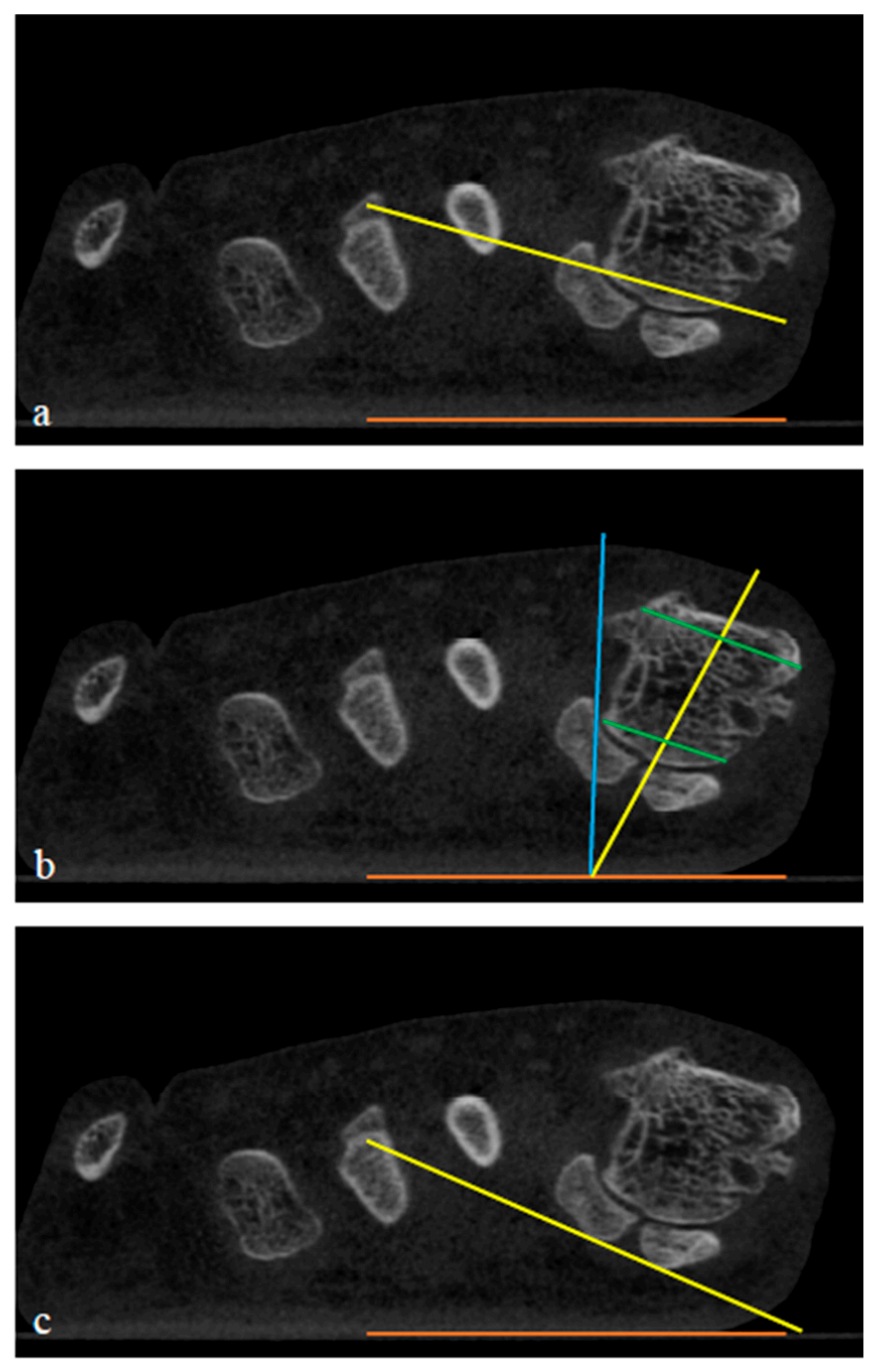
Rotational assessment of the first ray on coronal weight-bearing CT (WBCT) images. The metatarsal pronation angle (MPA) (**a**) is formed by a line connecting the inferomedial and inferolateral margins of the plantar sesamoid facets of the first metatarsal and a horizontal line parallel to the weight-bearing plane. The alpha angle (**b**) is determined using lines drawn through the medial and lateral margins of the metatarsal head and the borders of the plantar grooves, with the vertical axis serving as the reference. The sesamoid rotation angle (**c**) corresponds to the inclination of the line connecting the plantar surfaces of the medial and lateral sesamoids relative to the weight-bearing plane. In all panels, the orange line represents the horizontal weight-bearing reference plane, while the yellow lines indicate the anatomical orientations used for angular measurements. In panel (**b**), the blue line represents the vertical reference axis, and the green lines indicate the anatomical landmarks used to define the alpha angle.

In the normal foot, first-metatarsal rotation remains limited and permits a functional relationship among the metatarsal head, sesamoids, and supporting surface [30,31]. The alpha angle, metatarsal pronation angle (MPA), and sesamoid rotation angle quantify complementary components of this orientation on coronal WBCT images (Figure 1) [4,5,32]. These rotational parameters should not be considered interchangeable because they characterize rotation at different anatomical levels and depend on distinct landmarks and reference axes. Their reported values may also vary with image acquisition, foot positioning, segmentation, and measurement methodology. Accordingly, parameter-specific reproducibility and methodological consistency should be considered when comparing values across studies or applying proposed reference thresholds to individual patients. In asymptomatic individuals, these measurements generally indicate limited first-metatarsal pronation, although reported values vary according to the anatomical landmarks and measurement method used [4,5,21].

This control also depends on muscular structures. The fibularis longus contributes to plantar support of the first ray, whereas the tibialis anterior and intrinsic muscles help maintain its orientation and stabilize the hallux [10]. These muscular contributions form part of the biomechanical interpretation of first-ray function but are not directly assessed by static WBCT [10].

### 4.3. Metatarsosesamoid Congruity and Load Transmission

The sesamoids, which are embedded within the plantar plate, guide MTP1 motion and distribute the loads applied to the first-metatarsal head [10]. The sagittal crista and plantar facets help maintain their trajectory during hallux dorsiflexion [33].

The sesamoid complex functions as a pulley system that increases the moment arms of the flexor tendons and facilitates force transmission during propulsion [11,34]. In the normal foot, the sesamoids remain centered beneath the metatarsal head and maintain congruent articular relationships [4,35]. WBCT enables assessment of their position and rotation, as well as the coverage of the articular surfaces [2,35,36].

### 4.4. Windlass-Mediated Stiffening and Propulsion

Hallux dorsiflexion tensions the plantar aponeurosis, elevates the medial longitudinal arch, and increases foot stiffness during propulsion [13]. The effectiveness of this mechanism depends on sufficient MTP1 mobility, preserved metatarsosesamoid congruity, and a stable medial column [10,30].

Normal first-ray function therefore depends on the integration of these four properties. These biomechanical principles provide the functional framework for interpreting the structural abnormalities observed on WBCT in HV; however, the imaging findings themselves should not be considered direct measurements of dynamic first-ray function.

## 5. Biomechanical Interpretation of Structural WBCT Abnormalities in Hallux Valgus

The principal structural abnormalities identified by WBCT in hallux valgus—including altered first tarsometatarsal relationships, first-metatarsal pronation, metatarsosesamoid dissociation, and medial-column malalignment—have been extensively characterized in the imaging literature [2,4,19,20,21,35,37]. Rather than reiterating their role as three-dimensional imaging descriptors, this section examines how these structural observations may be interpreted in relation to first-ray biomechanics. For each abnormality, the distinction is maintained between what WBCT directly demonstrates under static loading conditions, what can reasonably be interpreted from established anatomical or biomechanical evidence, and what remains unproven regarding dynamic function or clinical outcome.

### 5.1. Tarsometatarsal Alignment Under Load and Its Biomechanical Interpretation

**Figure 2:**
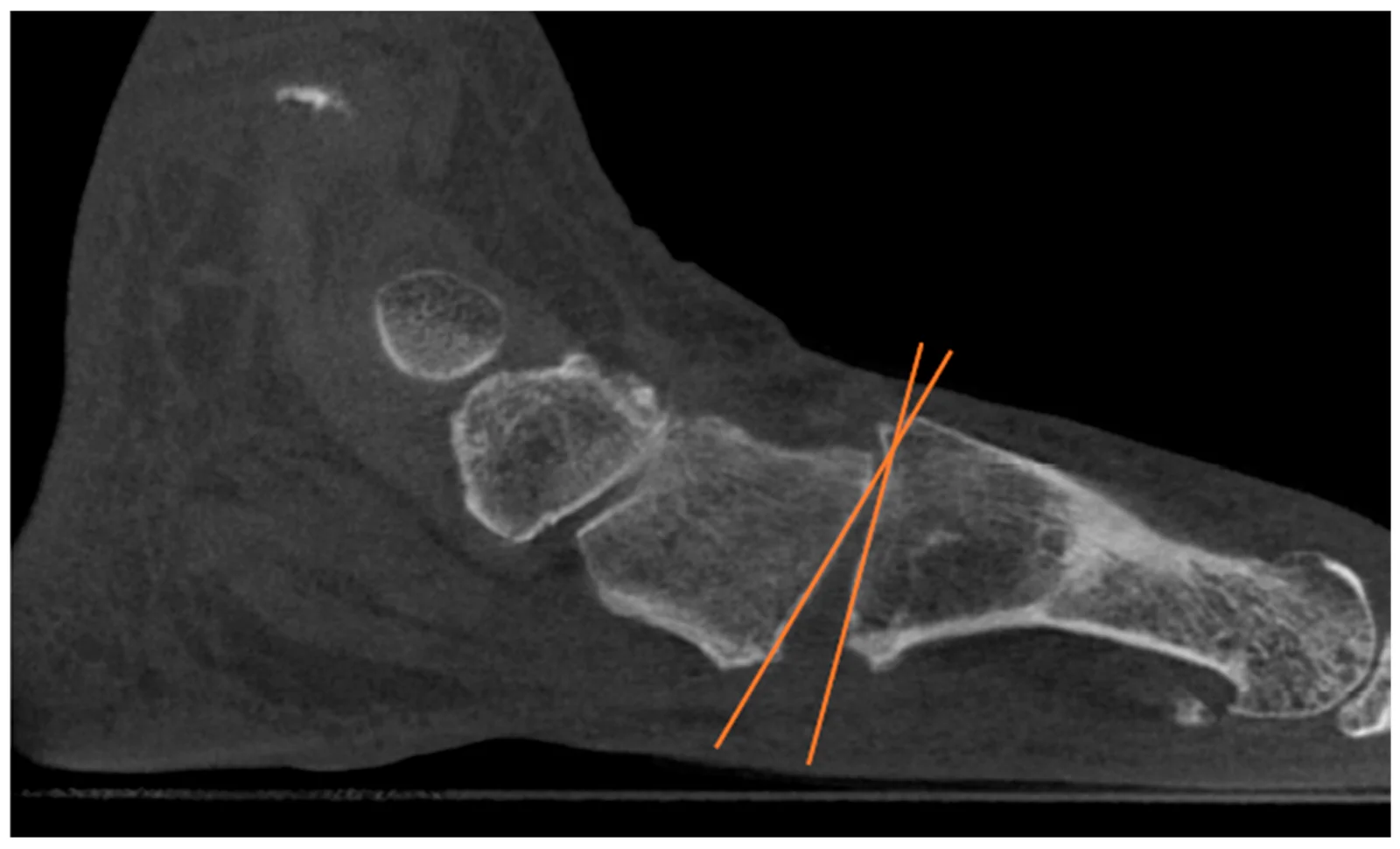
Measurement of the tarsometatarsal angle between the medial cuneiform and the first metatarsal. This angle is formed by two lines tangential to the articular surfaces of the medial cuneiform (C1) and the base of the first metatarsal (M1), drawn at the midpoint of the tarsometatarsal joint along its mediolateral axis.

Three-dimensional and weight-bearing imaging studies demonstrate altered spatial relationships at the first tarsometatarsal joint in hallux valgus, including changes in C1–M1 alignment and displacement under load (Figure 2) [5,21,38,39,40]. These findings constitute structural evidence of abnormal tarsometatarsal configuration rather than direct measurements of mechanical instability.

From a biomechanical perspective, the first tarsometatarsal joint contributes to the balance between adaptive first-ray mobility and maintenance of an effective orientation under load [10,12,41]. Altered C1–M1 alignment or displacement may therefore be interpreted as potentially affecting the mechanical support provided by the medial column and the spatial orientation of the first metatarsal. Such an interpretation is consistent with anatomical and experimental concepts of first-ray function, but static WBCT cannot determine whether the observed configuration reflects abnormal dynamic motion, altered ligamentous restraint, or impaired muscular control.

Accordingly, the term “instability” should be used cautiously when derived from a single static WBCT acquisition. Unless comparative loading or motion is directly assessed, WBCT demonstrates alignment and displacement under a specified loading condition; the functional consequences of these findings require corroboration by dynamic, mechanical, or clinical evidence.

### 5.2. First-Metatarsal Rotation: Structural Finding and Biomechanical Significance

First-metatarsal pronation is a reproducible three-dimensional feature of hallux valgus that can be characterized on WBCT at different anatomical levels [4,5,21,32]. As detailed above, the available rotational parameters characterize complementary anatomical relationships. In this section, these measurements are considered primarily as structural descriptors rather than as independent markers of biomechanical dysfunction.

The biomechanical significance of first-metatarsal rotation derives from its relationship to the orientation of the metatarsal head within the metatarsophalangeal–sesamoid complex. Changes in rotational orientation may alter the spatial relationship between the metatarsal head, sesamoids, and periarticular soft-tissue structures and may consequently modify tendon moment arms and force vectors around MTP1 [4,10,11,42,43]. These mechanical consequences are supported by anatomical and biomechanical principles but are not directly quantified by static WBCT.

Importantly, the first ray should not be assumed to rotate as a single rigid segment. Available three-dimensional studies indicate that rotational differences may occur at multiple levels, including the tarsometatarsal joint, metatarsal shaft, and metatarsal head [20,21]. The magnitude and distribution of rotation therefore vary among patients, reinforcing the interpretation of pronation as one component of a heterogeneous three-dimensional configuration rather than as a single causal mechanism of hallux valgus.

### 5.3. Metatarsosesamoid Relationships: From Structural Dissociation to Mechanical Interpretation

WBCT demonstrates that metatarsosesamoid malalignment in hallux valgus reflects a three-dimensional alteration in the relationship between the first-metatarsal head and the sesamoid complex rather than simple lateral displacement of the sesamoids [2,4,35]. Because the sesamoids remain relatively constrained by the plantar soft-tissue apparatus while the first metatarsal translates and rotates, WBCT can directly characterize structural dissociation and loss of articular congruity under load.

The biomechanical relevance of this structural relationship derives from the role of the sesamoid complex in guiding MTP1 motion, transmitting load, and increasing the moment arms of the plantar flexor structures [10,11,34]. Altered metatarsosesamoid congruity may therefore modify the mechanical environment of MTP1 and potentially influence force transmission during propulsion. However, WBCT demonstrates the geometry of this relationship rather than the resulting tendon forces, contact pressures, cartilage loading, or dynamic joint behavior.

Metatarsosesamoid dissociation should therefore be interpreted as a structural finding with plausible biomechanical consequences rather than as a direct measure of functional impairment. This distinction is particularly important when articular coverage or distance-mapping methods are used: these techniques quantify geometric relationships between surfaces but do not, by themselves, establish abnormal pressure distribution or impaired propulsion [36].

### 5.4. Medial-Column Coupling: Structural Associations and Limits of Functional Inference

WBCT studies demonstrate that first-ray abnormalities may coexist with rotational and alignment changes elsewhere in the medial column and hindfoot [19,20,22,23,44]. These observations support the concept that the first ray functions within an interconnected segmental system rather than as an isolated structure. Importantly, however, the distribution of these abnormalities is heterogeneous: hallux valgus may remain predominantly localized to the first ray, whereas more extensive medial-column involvement may occur in association with flatfoot or progressive collapsing foot deformity [22,23,44].

From a biomechanical perspective, these structural associations are compatible with segmental coupling, whereby changes in proximal alignment may influence distal first-ray orientation and vice versa. Nevertheless, cross-sectional WBCT cannot determine the direction of this relationship or establish whether hindfoot or medial-column alignment causes, compensates for, or merely coexists with first-ray deformity. The term “coupling” should therefore describe an anatomical and biomechanical association rather than an established causal pathway.

This distinction illustrates a broader limitation of static three-dimensional imaging: simultaneous abnormalities across several segments can define a structural pattern under load, but they do not establish the temporal sequence or dynamic mechanism through which that pattern developed. Normal first-ray functions and the corresponding structural abnormalities relevant to this interpretative framework are summarized in Table 1.

## 6. An Interpretative Framework Linking Structure, Biomechanics, and Function

The principal value of integrating WBCT findings is not to establish a new sequence of hallux valgus pathogenesis, but to distinguish how structural observations at different anatomical levels may be interpreted within the mechanics of the first ray. Altered TMT1 relationships, first-metatarsal rotation, metatarsosesamoid dissociation, and medial-column malalignment may coexist under load [2,4,19,20,21]. Their coexistence supports an integrated interpretation, but does not demonstrate that one abnormality necessarily precedes or causes another.

Within this framework, structural WBCT findings constitute the first level of evidence. Anatomical and experimental knowledge provides a second level by identifying plausible mechanical consequences of altered alignment or congruity, including changes in joint relationships, tendon moment arms, segmental orientation, or load transmission [9,10,11,12,13,42,43]. A third level concerns dynamic function and clinical outcome. These consequences cannot be established from static WBCT alone and require evidence from gait analysis, plantar-pressure assessment, experimental biomechanics, longitudinal imaging, or patient-reported outcomes.

This hierarchy is particularly relevant when several abnormalities coexist. For example, first-metatarsal pronation and metatarsosesamoid dissociation may be structurally associated, but their coexistence does not determine which developed first. Similarly, associations between hindfoot alignment, medial-column rotation, and first-ray orientation support segmental coupling without establishing the direction of mechanical influence [19,20,22,23,44]. Cross-sectional WBCT therefore provides a spatial representation of a loaded configuration rather than a temporal model of deformity progression.

The term “biomechanical continuum” is retained only as a conceptual description of potentially interrelated abnormalities within the first-ray–medial-column system. It should not be interpreted as a validated pathophysiological sequence or as evidence that static structural abnormalities directly represent functional impairment. This distinction defines the principal interpretative boundary of WBCT: it can characterize three-dimensional morphology under load, whereas direct evaluation of force, stress distribution, joint loading, and dynamic motion requires complementary biomechanical methods [26,45].

**Figure 3:**
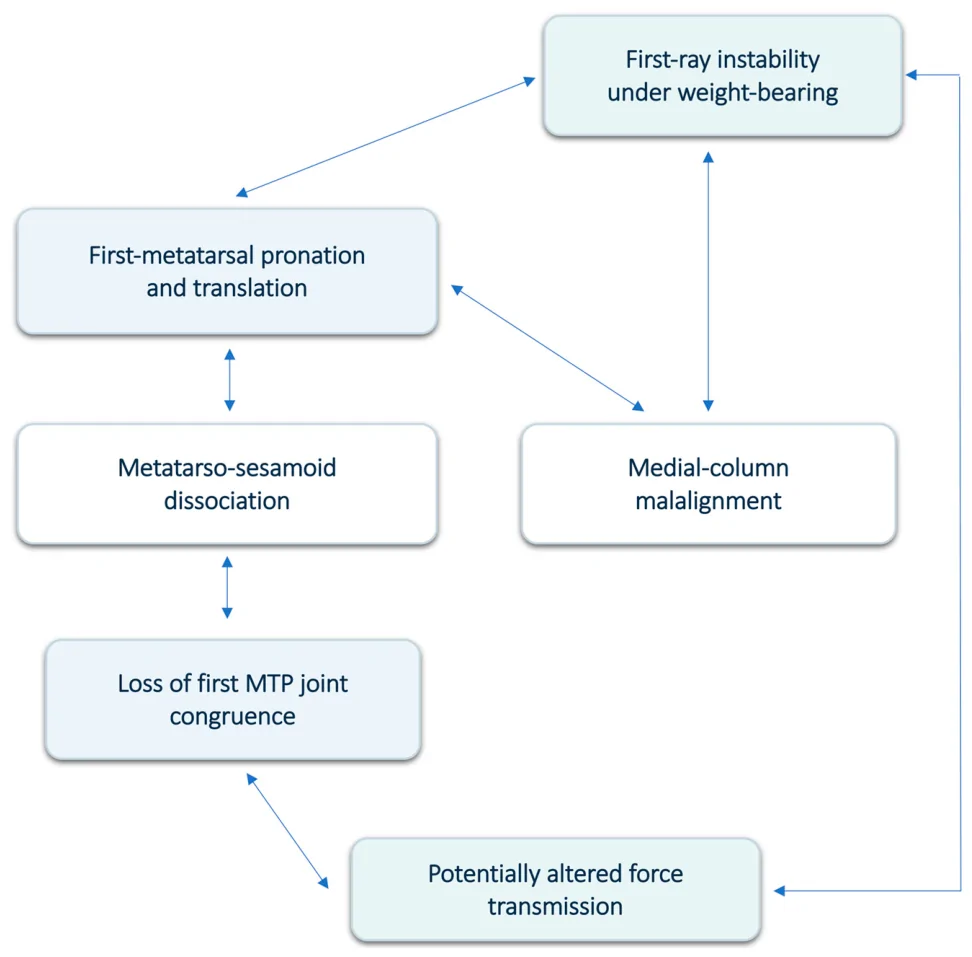
Integrated biomechanical continuum of hallux valgus. Conceptual representation of the main three-dimensional abnormalities identified by weight-bearing computed tomography in hallux valgus and their potential biomechanical relationships. Altered first-ray alignment under load, first-metatarsal pronation and translation, metatarsosesamoid dissociation, loss of congruence of the first metatarsophalangeal joint, and medial-column malalignment may coexist and potentially interact. Bidirectional arrows represent potential associations within this conceptual framework and imply neither a mandatory sequence of progression nor a universal causal relationship. MTP1, first metatarsophalangeal joint.

Figure 3 summarizes this structure-to-function framework and emphasizes the separation between directly observed WBCT abnormalities, their evidence-supported biomechanical interpretation, and functional consequences that remain incompletely established.

## 7. Structural Correction Versus Functional Restoration: Interpretative Limits of WBCT

### 7.1. What WBCT Can Demonstrate After Correction

Postoperative WBCT can characterize residual or corrected three-dimensional structural relationships that may not be fully represented by conventional angular measurements. These include first-metatarsal rotational orientation, metatarsosesamoid congruity, TMT1 alignment, and segmental relationships within the medial column [32,46,47,48,49,50,51]. Accordingly, normalization of the hallux valgus angle or intermetatarsal angle should not be assumed to represent normalization of all three-dimensional components of the deformity.

Different procedures may produce different structural changes. Residual first-metatarsal pronation has been reported after osteotomy, whereas procedures involving the first tarsometatarsal joint may modify transverse alignment, rotational orientation, sesamoid position, and proximal first-ray relationships [32,46,49,50,51]. These observations are useful for characterizing the three-dimensional result of correction, but they should not be interpreted as evidence that one structural pattern or surgical technique is universally biomechanically superior.

Quantitative rotational parameters may assist in describing residual or corrected first-metatarsal orientation. An alpha-angle value of approximately 15.8° has been proposed in an individual study as a threshold for identifying increased first-metatarsal pronation [4]. However, reported rotational values vary substantially according to the measurement method, reference landmarks, loading conditions, and study population [4,52]. Accordingly, such values should be regarded as study-specific reference thresholds rather than validated clinical or surgical cutoffs. Their interpretation depends on the population studied, anatomical landmarks, imaging system, and measurement technique, and exceeding a particular threshold does not independently establish a specific pathological mechanism or determine the appropriate surgical procedure.

### 7.2. Why Structural Normalization Does Not Establish Functional Restoration

The distinction between structural correction and functional restoration is central to the interpretation of postoperative WBCT. Improved rotational alignment or metatarsosesamoid congruity may plausibly contribute to a more favorable mechanical configuration of the first ray, but static imaging does not directly demonstrate normalization of tendon forces, joint loading, dynamic stability, gait mechanics, or propulsive efficiency. Structural improvement should therefore be regarded as an imaging outcome rather than as a surrogate for functional recovery unless an independent relationship has been demonstrated.

Residual first-metatarsal pronation after conventional angular correction is clinically relevant because satisfactory correction in the transverse plane may coexist with persistent rotational malalignment [32,47,48,51]. Conversely, procedures that modify the first tarsometatarsal relationship may produce correction across several planes [32,49]. These observations support postoperative assessment of rotational and articular relationships in addition to conventional angular correction. However, they demonstrate differences in structural correction rather than direct restoration of dynamic first-ray biomechanics.

Associations between greater correction of first-metatarsal pronation, lower recurrence, and improved patient-reported outcomes have been reported [50]. However, observational associations do not establish that normalization of an individual WBCT parameter independently causes superior clinical outcomes. Similarly, residual three-dimensional abnormalities may coexist with recurrence, but they should not automatically be interpreted as its sole biomechanical cause.

WBCT should therefore complement rather than replace symptoms, physical examination, conventional weight-bearing radiographs, and functional assessment. An abnormal postoperative WBCT parameter does not by itself constitute an indication for additional surgery, and no individual three-dimensional measurement currently functions as a universally validated surgical target or stand-alone decision criterion.

The principal WBCT parameters used to describe structural correction in hallux valgus and their interpretative limitations are summarized in Table 2.

**Table 2:** Selected Three-Dimensional WBCT Parameters for Describing Structural Correction in Hallux Valgus and Their Interpretative Limitations.

Domain	WBCT Assessment	Potential Role In Assessment of Correction
Malalignment	HVA and IMA	Assessment of transverse-plane angular correction
M1 rotation	Alpha angle and MPA	Assessment of rotational correction
TMT1 relationship	C1–M1 relationship and dorsal translation	Assessment of residual TMT1 malalignment or displacement under load
Sesamoid complex	Position, rotation, and articular coverage	Assessment of sesamoid recentering and metatarsosesamoid congruity
Medial column	Segmental alignment under load	Assessment of residual segmental malalignment

Note: These WBCT parameters complement clinical and conventional radiographic assessment but do not constitute universally validated surgical targets or stand-alone decision-making criteria. Static WBCT findings should not be interpreted as direct measurements of physiological mobility, force transmission, or functional recovery. Abbreviations: C1, medial cuneiform; HVA, hallux valgus angle; IMA, intermetatarsal angle; M1, first metatarsal; MPA, metatarsal pronation angle; TMT1, first tarsometatarsal joint; WBCT, weight-bearing computed tomography.

## 8. Bridging the Gap Between Structural Imaging and Functional Biomechanics

The principal limitation of WBCT for biomechanical interpretation is that it provides three-dimensional geometry under a defined static loading condition but does not directly measure the forces responsible for that configuration. The next methodological step is therefore not simply to generate additional imaging parameters, but to integrate structural information with complementary methods capable of assessing mechanics, motion, and clinical function.

Quantitative tools such as automated segmentation and articular mapping may improve the reproducibility and spatial characterization of WBCT-derived anatomy [24,36]. Their contribution, however, remains primarily structural. Distance and coverage maps quantify relationships between articular surfaces but do not directly measure cartilage loading, contact pressure, or dynamic joint mechanics. Similarly, artificial-intelligence methods may facilitate automated extraction of imaging features, but current evidence in hallux valgus is substantially more mature for conventional radiographs than for volumetric WBCT datasets [53,54]. AI-based WBCT analysis should therefore be considered an emerging technical approach rather than an established method of functional assessment.

### 8.1. From Patient-Specific Geometry to Mechanical Simulation

Finite element analysis (FEA) provides one potential means of extending WBCT-derived geometry toward the estimation of mechanical behavior. Patient-specific three-dimensional anatomy can serve as the geometrical basis of computational models designed to estimate stress, contact mechanics, and load transmission [45,55]. In this context, WBCT and FEA provide fundamentally different but potentially complementary information: WBCT characterizes structural configuration under load, whereas FEA estimates mechanical responses according to specified model assumptions.

Anatomical realism, however, should not be equated with biomechanical validity. FEA outputs depend on the assigned material properties, representation of cartilage and ligaments, muscular forces, contact definitions, boundary conditions, and simulated loading conditions. A patient-specific WBCT geometry can therefore improve anatomical representation without guaranteeing that the resulting stress or pressure estimates reproduce in vivo mechanics. Experimental validation and correlation with clinical or functional measurements remain necessary before such models can support individual treatment decisions.

This distinction illustrates the broader structure-to-function framework of this review: increasingly sophisticated structural models may improve biomechanical hypotheses, but functional validity requires independent evidence beyond imaging geometry alone.

### 8.2. Functional Validation as the Next Research Priority

The critical next step for WBCT research in hallux valgus is to determine which structural parameters have reproducible relationships with independently measured function and clinical outcome. This will require studies combining standardized WBCT acquisition with gait analysis, plantar-pressure assessment, validated computational modeling, patient-reported outcomes, and longitudinal follow-up.

Longitudinal designs are particularly important because cross-sectional associations cannot determine whether a structural abnormality precedes disease progression, represents compensation, or simply coexists with other components of the deformity. Similarly, postoperative normalization of a WBCT parameter should not be regarded as a validated surrogate endpoint until its relationship with recurrence, symptoms, function, or patient-reported outcomes has been independently demonstrated.

The future value of WBCT will therefore depend less on increasing the number of available measurements than on establishing which structural findings are reproducible, biomechanically meaningful, and clinically predictive.

## 9. Conclusions

WBCT provides a three-dimensional structural characterization of hallux valgus under static weight-bearing conditions, revealing rotational, articular, and segmental relationships that are incompletely represented by conventional angular measurements. Its principal biomechanical value lies not in directly measuring function but in providing anatomically detailed structural observations that can be interpreted in the context of established first-ray mechanics.

Interpretation of WBCT findings is also limited by methodological variability across studies, including differences in scanner technology, unilateral versus bilateral weight-bearing, load distribution between limbs, lower-limb positioning, segmentation, anatomical landmark selection, and measurement technique. These factors may affect quantitative measurements and currently limit direct comparison across studies and the definition of universal reference values.

A critical distinction should therefore be maintained between structural findings demonstrated by WBCT, their biomechanical interpretation, and functional or prognostic consequences requiring independent validation. Future studies combining standardized WBCT with dynamic biomechanical assessment and longitudinal clinical outcomes are needed to determine which three-dimensional findings provide reproducible functional or prognostic information.

## Figures and Tables

**Table 1:** From Structural WBCT Findings to Biomechanical and Functional Interpretation in Hallux Valgus.

Domain	Structural Finding Assessed by WBCT	Biomechanical Interpretation	Functional Inference and Current Limitation
TMT1 relationship	Altered C1–M1 alignment and displacement under load	May reflect altered mechanical support and orientation of the first ray	Static alignment does not directly demonstrate dynamic instability
First-metatarsal rotation	Increased or altered rotational orientation	May modify metatarsal-head orientation and periarticular force vectors	Does not directly quantify muscular forces or dynamic rotational control
Metatarsosesamoid relationship	Dissociation, rotation, or reduced articular congruity	May alter the mechanical relationship between the metatarsal head and sesamoid apparatus	Does not directly measure tendon moment arms, contact pressure, or propulsive efficiency
Medial-column relationship	Segmental rotational or alignment abnormalities	May reflect intersegmental mechanical coupling	Cross-sectional WBCT cannot establish direction of causality or temporal sequence
Multisegment configuration	Coexisting first-ray, medial-column, and hindfoot abnormalities	May represent an integrated load-dependent structural pattern	Does not establish a universal biomechanical pathway or predict functional outcome

Note: WBCT provides three-dimensional structural information under static weight-bearing conditions. Biomechanical interpretations require support from anatomical, experimental, or mechanical evidence, whereas dynamic functional and prognostic consequences require independent validation. C1, medial cuneiform; M1, first metatarsal; TMT1, first tarsometatarsal joint; WBCT, weight-bearing computed tomography.

## Data Availability

No new data were created or analyzed in this study. Data sharing is not applicable to this article.
